# Prevalence of latent tuberculosis in homeless persons: A single-centre cross-sectional study, Germany

**DOI:** 10.1371/journal.pone.0214556

**Published:** 2019-03-26

**Authors:** Friederike von Streit, Christoph Bartels, Thorsten Kuczius, Christoph Cassier, Joachim Gardemann, Frieder Schaumburg

**Affiliations:** 1 Institute of Medical Microbiology, University Hospital Münster, Münster, Nordrhein-Westfalen, Germany; 2 Klinik am Schlossgarten Dülmen, Dülmen, Nordrhein-Westfalen, Germany; 3 Institute of Hygiene, University Hospital Münster, Münster, Nordrhein-Westfalen, Germany; 4 Abteilung Ärztliche Gutachten und Abteilung Infektionsschutz und Umwelthygiene, Amt für Gesundheit, Veterinär- und Lebensmittelüberwachung, Münster, Nordrhein-Westfalen, Germany; 5 Kompetenzzentrum Humanitäre Hilfe, Fachhochschule Münster, Münster, Nordrhein-Westfalen, Germany; University of Cape Town, SOUTH AFRICA

## Abstract

**Purpose:**

Homeless persons have a high risk for tuberculosis. The prevalence of latent tuberculosis infection and the risk for a progression to active tuberculosis is higher in the homeless than in the general population. The objective was to assess the prevalence and risk factors of tuberculosis/latent tuberculosis infection in a homeless population in Germany.

**Methods:**

Homeless individuals (n = 150) were enrolled in a cross-sectional study at three shelters in Münster, Germany (October 2017–July 2018). All participants were screened using an ELISPOT interferon-γ release assay (IGRA). Those participants tested positive/borderline by IGRA provided three sputa for microbiological analysis (line probe assay, microscopy, culture) and underwent a chest X-ray to screen for active pulmonary TB. Risk factors for tuberculosis/latent tuberculosis infection were analysed using a standardized questionnaire.

**Results:**

Of the 142 evaluable IGRA, 21 (15%) were positive and two (1%) were borderline. No participant with a positive/borderline IGRA had an active tuberculosis as assessed by chest X-ray and microbiology. A negative IGRA was associated with a citizenship of a low-incidence country for tuberculosis (according to WHO, p = 0.01), low-incidence country of birth (p<0.001) or main residence in a low-incidence country in the past five years (p = 0.002).

**Conclusions:**

The prevalence of latent tuberculosis infection (diagnosed by a positive/borderline IGRA) was 16%; no active tuberculosis was detected. The highest risk for latent tuberculosis infection was found in patients from high-incidence countries. This population at risk should be either treated for latent tuberculosis infection or need to be monitored to early detect a progression into active disease.

## Introduction

The incidence of tuberculosis (TB) decreased in Germany in the past decades, but increased again between 2013 and 2016 (from 5.4 to 7.2 per 100,000/year) with 69% of notified patients being non-German citizens [1]. The resurgence of TB carries a risk not only for the health of individuals and the population, but also for the country and its economy. The mean direct treatment cost of one adult case with an active TB is approximately 7,400 € and 52,300 € for infections with susceptible and multi-drug resistant *Mycobacterium tuberculosis*, respectively (excluding costs for medical care, loss of productivity, contact tracing) [2].

One major source sustaining the global TB epidemic are latent TB infections (LTBI) [3]. LTBI is defined by World Health Organization (WHO) as *“*A state of persistent immune response to stimulation by *M*. *tuberculosis* antigens with no evidence of clinically manifest active TB” [4]. The average risk of progression from LTBI to active TB during lifetime is 10% [5]. The most affected are immunocompromised persons (e.g. HIV infection, treatment with TNF-α inhibitors) and other at-risk groups (e.g. prisoners, homeless persons, illicit drug users) [4]. However, the exact prevalence of LTBI is mostly unknown for the majority of countries. Extrapolations from epidemiological studies suggest, that about one quarter of the global population has LTBI [6].

The diagnostic tools to detect LTBI are imperfect [7]. However, compared to the Tuberculosis Skin Test (TST), interferon-γ release assays (IGRA), especially the T-SPOT.TB, have a higher sensitivity (84% vs. 67%) and specificity (75% vs. 63%) to detect TB [8]. The IGRA is also considered to be superior to TST as it does not show false positive results in BCG-vaccinated persons and requires a single patient contact only [8,9].

Exposure to communicable diseases is especially high in the homeless population due to close contacts to large and fluctuating communities in shelters [10]. Consequently, the risk of hospitalization due to infections (including TB) is higher in homeless persons than in the general population [11]. This is in line with a high prevalence of positive IGRA in homeless persons in Poland (36.7%), USA (12–40%), Japan (50.6%) and South Korea (75.9%) in non-outbreak settings [12–16].

LTBI is found more often in homeless living under crowded conditions than in those sleeping rough (33–45% vs. 23%) [17]. Besides the close contact, the late state of disease at diagnosis is an important risk factor for ongoing transmission between homeless persons [18]. Noteworthy, the incidence rate of active TB in persons with positive IGRA is higher in homeless (30/1000 person-years) compared to close contacts to index cases (18/1000 person-years) [12]. Although homeless persons have a high risk of TB, the exact prevalence of LTBI in this population in Germany is unknown. Therefore, the objective of our study was to assess the prevalence and risk factors of TB/LTBI in homeless individuals.

## Methods

### Ethics approval and consent to participate

Ethical approval was obtained from the ethical committee of the Medical Association of Westfalen-Lippe and the University of Münster (2017-349-f-S).

All participants gave a written informed consent prior to any study related activities. All procedures were conducted in cooperation with the local Public Health Agency (Gesundheitsamt der Stadt Münster, Münster, Germany).

### Participants

Considering an expected prevalence of 36% of LTBI among homeless persons (as shown in a comparable population in Poland) [12], a 95% confidence interval of 25–47% and α = 0.05, the expected population size is 146. To account for non-interpretable IGRA results, we aimed to enrol 150 participants.

Participants were recruited between October 2017 and July 2018 at three shelters for homeless persons in Münster, Germany (“Haus der Wohnungslosenhilfe” (accommodation and support for men), “Gertrudenhaus” (accommodation and support for women), “Treffpunkt an der Clemenskirche” (supply of meals and support)). To improve compliance, we worked closely with the existing social and health care services. Criteria for inclusion were (i) written informed consent, (ii) use of assistance for homeless people provided by one of the three shelters mentioned above, (iii) an age of ≥18 years and (iv) proficiency in one of the languages, which were used for the informed consent and questionnaire (i.e. German, Arabic, Bulgarian, Hungarian, Polish, Russian, Slovakian, English, S1 and S2 File). The only exclusion criterion was a known pregnancy since chest X-ray had to be done for all IGRA positive cases according to the protocol. A compensation for participation (vouchers for three meals or 5 €) was offered.

A questionnaire was completed by each participant with assistance of the study personnel. It included demographic data (age, sex, overall duration of homelessness, duration of stay at shelter, country of birth, citizenship, main residence in the past five years, former and current employment), medical history (chronic diseases, regular medication, addictions), as well as TB-specific information (BCG vaccination, contact to infected persons, history and symptoms of TB). All symptoms were recorded as reported by the participants and assessed by a physician.

**Fig 1:**
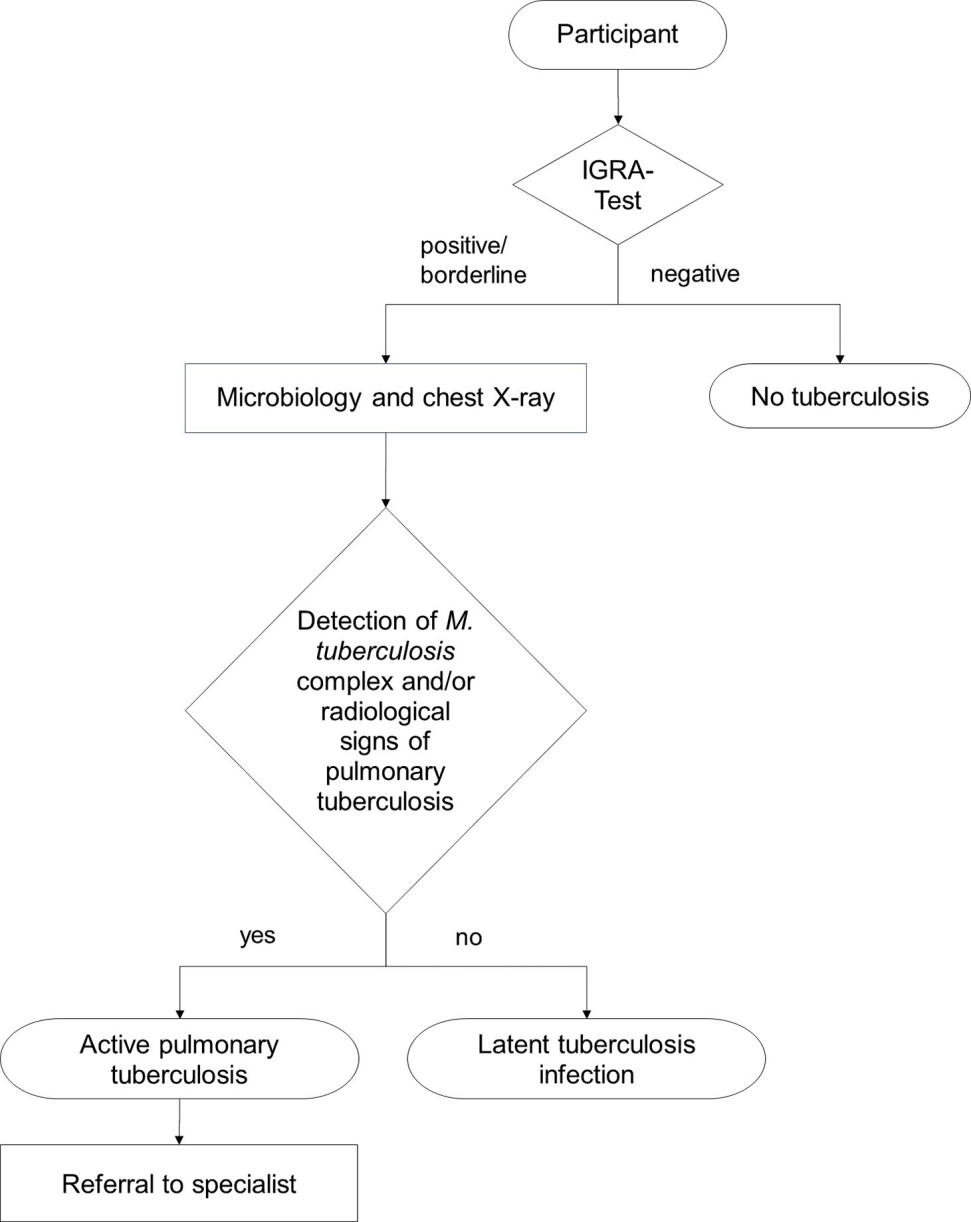
Diagnostic procedures to determine tuberculosis (TB) infections in participants. All Participants were tested by Interferon-gamma release assay (IGRA). Patients with borderline/positive IGRA results were screened for active pulmonary TB. Latent TB infection (LTBI) was defined as “a state of persistent immune response to stimulation by *Mycobacterium tuberculosis* antigens with no evidence of clinically manifest active TB” according to the World Health Organization [4].

The TB incidence of countries were taken from the WHO’s Global tuberculosis report [19]. Active pulmonary TB was diagnosed if *M*. *tuberculosis* was detected in sputum samples or if chest X-ray showed any typical signs for active TB in IGRA positive individuals (Fig 1).

### Microbiology

Blood samples (Lithium-Heparin-Tube) were stored at room temperature (18–25°C) for a maximum of 32 hours until the IGRA (T-SPOT.TB, Oxford Immunotec, Oxfordshire, UK) was performed and interpreted according to the manufacturer’s instructions; “T-cell Xtend” solution was used if the samples were not processed within eight hours after sampling. The number of spots in the nil control was subtracted from the number of spots following the stimulation of interferon-γ secretion with *M*. *tuberculosis* antigens (ESAT-6 and/or CFP10). Based on the difference of the number of spots, test results were interpreted as negative (≤4 spots), borderline (5–7 spots) or positive (≥8 spots). In case of positive or borderline results, further diagnostics were initiated to identify potential cases of active pulmonary tuberculosis (Fig 1). A chest X-ray was performed and interpreted by a specialist in medical radiology. Three independent sputum samples (e.g. taken on three consecutive days) were analysed as recommended by WHO [9]. Ziehl-Neelsen stained sputa were screened for acid-fast bacilli by light microscopy. In parallel, sputa were tested by a line-probe assay to screen for *M*. *tuberculosis* complex and determinants for rifampicin or isoniazid resistance (GenoType MTBDRplus, Hain Lifescience, Nehren, Germany). Specimens were also cultured both in liquid media (BACTEC MGIT, BD Diagnostic Systems, Heidelberg, Germany) for maximum six weeks and on solid media (Lowenstein-Jensen agar, Stonebrink) for maximum eight weeks [9].

### Referral for therapy

All participants with positive findings in chest X-ray and/or sputum were referred to a specialist to undergo treatment (Fig 1). According to the German TB guideline, treatment of LTBI should be considered for patients with a high risk for reactivation (including homeless people) [20].

### Statistics

Calculations were performed with “R” (version 3.5.1, package “epiDisplay”). The significance level was set at α = 0.05. Categorical variables were compared between IGRA positive and IGRA negative individuals using chi^2^ test or Fisher’s exact test, where appropriate. Multivariable analysis was done with logistic regressions. Continuous values were tested for normal distribution by Shapiro-Wilk test; normally distributed variables were compared with Student’s t-test. Non-normal data were compared with Wilcoxon rank-sum test.

## Results

### Demographic data

Overall, 150 persons were included in the study. We were unable to draw blood samples (e.g. due to long-term intravenous drug abuse) in three cases and in five cases the IGRA yielded non-interpretable results (e.g. too few lymphocytes in blood, technical errors). These persons were excluded from the final analysis (n = 8). The per protocol population (n = 142) had a mean (SD) age of 42 years (12) and an imbalanced ration of females to males (14:128, Table 1). The calculated mean BMI (SD) was 25.2 kg/m^2^ (5.0; S1 Table).

**Table 1:** Characteristics of the study population that showed significant association with Interferon-γ release assay (IGRA) results. (Further recorded data shown in S1.).

		Total (n = 142)	IGRA negative (n = 119)	IGRA positive/borderline (n = 23)	OR (95%CI)	p-value
Demographic data	Mean age [years] (SD)	42.4 (12)	41.9 (12)	45.0 (15)	NA	0.4
	Sex [female]	14 (10)	12 (10.1)	2 (9)	0.9 (0.2–4.1)	1
	Health insurance	119 (84)	103 (87)	16 (70)	2.8 (1−7.9)	0.04
Citizenship	Bulgarian	9 (6)	4 (3)	5 (22)	Reference	-
	German	83 (58)	75 (63)	8 (35)	0.1 (0.02−0.4)	0.01
	Polish	12 (8)	10 (8)	2 (9)	0.2 (0.02−1.2)	0.1
	Slovak	9 (6)	7 (6)	2 (9)	0.2 (0.03−1.8)	0.2
	Other^a^	29 (20)	23 (19)	6 (26)	0.2 (0.04−1.0)	0.05
Country of birth	Bulgaria	9 (6)	4 (3)	5 (22)	Reference	-
	Germany	73 (51)	68 (57)	5 (22)	0.06 (0.01−0.3)	<0.001
	Poland	13 (9)	11 (9)	2 (9)	0.2 (0.02−1.1)	0.06
	Other^b^	47 (33)	36 (30)	11 (48)	0.2 (0.1−1.1)	0.06
Country of main residence in the past 5 years	Germany	114 (80)	98 (82)	16 (67)	0.04 (0−0.4)	0.005
	Bulgaria	5 (4)	1 (1)	4 (17)	Reference	-
	Other^c^	23 (16)	20 (17)	3 (13)	0.04 (0−0.5)	0.01
Exposure to TB^d^	Citizen of a low-incidence country	98 (70)	87 (74)	11 (48)	0.3 (0.1−0.8)	0.01
	Born in a low-incidence country	90 (64)	83 (70)	7 (30)	0.2 (0.1−0.5)	<0.001
	Main residence in a low-incidence country in the past 5 years	127 (93)	111 (96)	16(76)	0.1 (0.04−0.6)	0.002
Education	Median years of school attendance (range)	10 (0–20)	10 (0−20)	9 (0−12)	NA	0.006
Signs/symptoms of TB	Cough for more than 3 weeks	38 (27)	28 (24)	10 (44)	0.4 (0.2−1)	0.05

NB: All values are n (% of group–Total/IGRA negative/IGRA positive) unless indicated otherwise. NA = not applicable

^a^ Other citizenships: Austrian (n = 1), Congolese (n = 1), Croatian (n = 1) Ethiopian (n = 1), Greek (n = 1), Guinea-Conakrian (n = 1), Hungarian (n = 1), Iran (n = 1), Italian (n = 3), Kosovan (n = 1), Latvian (n = 1), Lithuanian (n = 2), Nigerian (n = 1), Portuguese (n = 1), Romanian (n = 3), Russian (n = 1), Senegalese (n = 1), Serbian (n = 1), Somalian (n = 1), Syrian (n = 2), Turkish (n = 3)

^b^ Other countries of birth: Azerbaidschan (n = 1), Congo (n = 1), Croatia (n = 1), Ethiopia (n = 1), Eritrea (n = 1), Gambia (n = 1), Greece (n = 1), Guinea-Conakry (n = 1), Hungary (n = 1), India (n = 1), Iran (n = 1), Iraq (n = 2), Israel (n = 1), Italy (n = 3), Kenia (n = 1), Kosovo (n = 1), Latvia (n = 1), Lithuania (n = 2), Netherlands (n = 1), Portugal (n = 1), Romania (n = 3), Russia (n = 1), Senegal (n = 1), Serbia (n = 1), Somalia (n = 1), Syria (n = 3), Turkey (n = 4)

^c^ Other countries of main residence in the past five years: Croatia (n = 1), Ethiopia (n = 1), travelled in European Union (n = 5), France (n = 1), Greece (n = 1), Hungary (n = 1), Italy (n = 2), Netherlands (n = 1), Poland (n = 1), Slovakia (n = 3), Spain (n = 1), Syria (n = 2), USA (n = 1), no data (n = 1)

^d^ Low-incidence countries for TB as defined by the World Health Organization (<100 cases per 100000 population) [4,19]

Of 142 volunteers, 21 (15%, 95%CI: 11–23) had positive and two (1%) had a borderline IGRA (5 and 7 spots). Patients with a positive or borderline IGRA were grouped together as the risk for a positive IGRA in re-tests can be up to 37% in high-risk groups [21].

The proportion of German citizens was higher in the IGRA negative group (63%, 75/119) compared to the IGRA positive/borderline group (35%, 8/23, p = 0.01, Table 1). We tested if a positive IGRA was not only associated with a German citizenship but also with persons born in Germany but with any citizenship (e.g. German and non-German). Here, the IGRA negative group comprised significantly more participants who were born in Germany (57%, 68/119) than in the IGRA positive group (22%, 5/23, p<0.001, Table 1). Similarly, those participants who resided mainly in Germany during the past five years were more likely IGRA negative (82%, 98/119) than IGRA positive (67%, 16/23 p = 0.005, Table 1).

Countries were stratified into low-incidence countries for TB as defined by WHO (<100 cases per 100,000 population) and high-incidence countries (all others) [4]. A negative IGRA was associated with a citizenship of a low-incidence country (IGRA negative vs. IGRA positive/borderline group: 74% (87/119) vs. 48% (11/23), p = 0.01), place of birth in a low-incidence country (70% (83/119) vs. 30% (7/32), p<0.001) and a main residence in a low-incidence country in the past five years (96% (111/119) vs. 76% (16/23), p = 0.002, Table 1).

The proportion of participants with a health insurance was higher in the IGRA negative compared to the IGRA positive group (87% (103/119) vs. 70% (16/23), OR = 2.8, 95%CI: 1−7.9, p = 0.04, Table 1).

IGRA negative participants had a longer school education (median of 10 years, range: 0−20) compared to IGRA positive participants (median of 9 years, range: 0−12, p = 0.006, Table 1).

Neither duration of homelessness nor incarceration was a risk factor for a positive IGRA (S1 Table).

### Medical information

Chronic cough (i.e. cough ≥3 weeks) was significantly associated with a positive IGRA (24% (28/119) vs. 44% (10/23), OR = 0.4, 95%CI: 0.2−1, p = 0.05, Table 1). Other signs and symptoms of TB (e.g. fever, night sweat, weight loss in the past three months, production of sputum when coughing) were not associated with a positive IGRA, (S1 Table). Noteworthy, no participant reported an HIV infection.

### Further consultation

Of the 23 participants with a positive/borderline IGRA, five were lost to follow-up for X-ray (22%) and seven were lost to follow-up for sputum analysis (30%). Of the remaining 18 participants, all underwent a chest X-ray to rule out active pulmonary TB. Except for one participant, no radiological signs of TB were seen (i.e. centrilobular nodules, tree-in-bud pattern, cavities, consolidations, tuberculoma, parenchymal scar, calcified hilar/paratracheal lymph nodes, traction bronchiectasis, fibrosis, pleural effusion [22]). The patient with CT signs of TB (i.e. tree-in-bud pattern) was hospitalized due to weight loss, night sweat and cough after inclusion. Bronchoscopy, microbiological analysis of sputum and bronchial exudate were performed and active TB was ruled out. Symptoms were most likely due to a culture confirmed *Haemophilus influenzae* pneumonia. None of the IGRA positive participants consented a preventive chemotherapy for LTBI.

In total, 47 sputum samples were analysed from 16 IGRA positive/borderline patients (median number of samples per patient 3; range: 2−3). All samples were negative by culture, microscopy and line-probe assay.

## Discussion

We performed a cross sectional study on the prevalence of TB/LTBI among homeless people and found 23 IGRA positive/borderline participants (16%). This proportion is lower than the overall estimation for LTBI prevalence in the world (23%, 95%CI: 20.4−26.4) [6]. Though a positive IGRA result does not necessarily mean a present infection with *M*. *tuberculosis*, it mirrors a lasting immune response to *M*. *tuberculosis*, and is considered a useful tool to detect LTBI (together with tuberculin skin test) [23].

Even though 27% of homeless persons in Germany are female, our sample only included 14 (10%) women [24]. The difference is due to the strategy of recruitment which was mainly performed at one shelter (“Haus der Wohnungslosenhife”) which provides housing only for men.

The prevalence of positive IGRA among homeless people was 36.7% in Poland, 12–32% in the USA, 50.6% in Japan and 75.9% in South Korea [12–16]. Compared to these studies, the prevalence in our population was rather low although the annual incidence of TB in Germany (7/100,000) is comparable with Poland (18/100,000), Japan (16/100,000) or the USA (3/100,000) [19]. A high risk of progression to active disease in the homeless makes our findings important for further policies and programmes to control TB in Germany [25]. This risk is supported by a high annual incidence of active pulmonary TB in homeless persons in Münster (2015: 270/100,000; 2016: 371/100,000; 2017: 126/100,000). These annual incidence is based on the number of notified TB cases (personal communication, C. Cassier) and the total number of homeless persons per year in Münster [26].

Our analysis showed that a citizenship of a low-incidence country, a place of birth or residence in a low-incidence country is associated with a negative IGRA (Table 1). This is in line with a declining annual incidence of active TB among German citizens between 2012 and 2016 (3.6 to 2.2 per 100,000) and increasing annual new cases of TB in non-German citizens (21.9 to 42.6 per 100,000) in Germany [1].

Screening for active TB in homeless in Frankfurt (Germany) found no difference in terms of the geographical origin of participants [27].

Presence of a health insurance was another item that showed a significant association with a negative IGRA. All German citizens are health insured by law. A health insurance can therefore be considered as a confounder of an association with a positive/borderline IGRA.

Our data suggest that lower educational levels (e.g. years of school attendance) increase the risk of a positive/borderline IGRA. This has already been described as risk factor for active tuberculosis in homeless [14]. It highlights the importance of education, including health issues, to prevent the spread of TB.

Five participants reported a known TB infection in the past (S1 Table). This could have been the primary TB, which developed later into LTBI in four participants [7]. The proportion of IGRA positive patients decreases during TB treatment (i.e. termination of exposure to *M*. *tuberculosis*) [28]. Therefore, the positive IGRA in participants with a self-reported TB infection in the past suggest that these participants have a true LTBI (i.e. constant exposure to *M*. *tuberculosis*).

Some limitations of our study need to be addressed. First, we only screened for active pulmonary TB in patients with positive/borderline IGRA. As 25% of TB cases are extrapulmonary, we might have missed these cases in our screening approach [1]. Second, participants were recruited at three shelters. Homeless persons without contact to supportive services and health care were not reached. Our population is therefore most likely not representative for the whole country. Third, the questionnaire was completed based on the information provided by the participant. We were unable to control particularly for a recall bias. Fourth, IGRA have a window period of approximately 4–7 weeks to become positive after exposure [29]. Therefore, there is a limited risk not to detect those participants with a very recent infection. Fifth, the exact prevalence of LTBI or positive IGRA results in the overall German population has not been investigated yet and we did not include a control group in our study. Therefore, we cannot deduce if the prevalence of LTBI is higher in the homeless group compared to the general population. Sixth, due to limited resources, we were unable to follow-up participants apart from referring them to specialists.

In conclusion, the prevalence of LTBI (diagnosed by a positive/borderline IGRA) was 16% in our population of homeless persons; no active TB was detected. The highest risk for LTBI had patients from high-incidence countries. This population at risk should be treated if LTBI is detected.

## Supporting information

S1 File Survey questionnaire German. (PDF)

S2 File Survey questionnaire English. (PDF)

S1 Table Characteristics of the study population and comparison of participants with and without a positive/borderline Interferon-γ release assay (IGRA). (DOCX)
