# Supplementary material for: Prevalence of latent tuberculosis in homeless persons: A single-centre cross-sectional study, Germany
Source: PLoS One. 2019 Mar 26;14(3):e0214556. doi: 10.1371/journal.pone.0214556 (PMC6435138; doi:10.1371/journal.pone.0214556)
Supplement: S1 File — (PDF) [file pone.0214556.s001.pdf]

Personalien

Getrennt von Studiendaten aufzubewahren

Studie: Tuberkulose bei Wohnungslosen

Häufigkeit der latenten Infektion mit *Mycobacterium tuberculosis* complex unter wohnungslosen Menschen in Münster

Studiennummer: MTBW ______

1. **Datum:**
2. **Person**
   a. Name, Vorname: ____
   b. Geburtsdatum: ___________________
   c. Kontakt im Fall positiver Testergebnisse:
      Telefon: ____
      Aufenthaltsort: ____

Case Report Form

Studie: Tuberkulose bei Wohnungslosen

Häufigkeit der latenten Infektion mit *Mycobacterium tuberculosis* complex unter wohnungslosen Menschen in Münster

Studiennummer: MTBW ______

EINSCHLUSSKRITERIEN

1. Einverständniserklärung
2. Mindestalter 18 Jahre
3. Erhaltene Einsichts- und Geschäftsfähigkeit
4. Proband spricht eine Sprache, in der die Studienunterlagen zur Verfügung stehen
5. Inanspruchnahme der Wohnungslosenhilfe in Münster

AUSSCHLUSSKRITERIEN

1. Ist bei Ihnen eine Schwangerschaft bekannt?

☐ Ja

☐ Nein

DEMOGRAPHISCHE DATEN

2. Datum:

3. Person

a. Geburtsjahr: ____________________
b. Geburtsort: _______________________
c. Nationalität: _______________________
d. Überwiegender Aufenthalt in den letzten 5 Jahren (Staat): _____________________
e. Gefängnisaufenthalt in der Vergangenheit:

☐ nein

☐ ja, Land: __________________

f. Bisherige Aufenthaltsdauer im HdW (Tage): _______

g. Geschlecht

( ) m ( ) w ( ) andere

h. Körpergröße (cm): _______________

i. Körpergewicht (kg): __________________

j. Ist eine BCG-Impfung bekannt?

( ) Ja ( ) Nein ( ) Unklar

4. Ausbildung/Beruf

a. Wieviel Jahre sind Sie zur Schule gegangen?

______ Jahre

b. Welchen Beruf üben Sie gerade aus

☐ ______________

☐ Ich bin zurzeit arbeitslos.

c. Welche Berufe haben Sie in der Vergangenheit ausgeübt?

____

____

5. Wie viele Monate waren Sie insgesamt in Ihrem Leben ohne festen Wohnsitz

☐ <6 Monate

☐ 6-24 Monate

☐ >24 Monate

6. Versicherungsstatus

☐ Gesetzliche Krankenversicherung in Deutschland

☐ Private Krankenversicherung in Deutschland

☐ Sozialamt

☐ In der EU versichert mit Leistungsansprüchen in Deutschland

☐ Im Ausland (außer EU) versichert mit Leistungsansprüchen in Deutschland

☐ Kein Versicherungsschutz

TUBERKULOSE

7. **Tuberkulose in der Vergangenheit**
    a. Wurde bei Ihnen in der Vergangenheit eine Tuberkuloseinfektion festgestellt?

    ( ) Ja ( ) Nein ( ) Unklar

    b. Wurden Sie in der Vergangenheit wegen einer Tuberkuloseinfektion behandelt?

    ( ) Ja ( ) Nein ( ) Unklar

    c. Hatten Sie in der Vergangenheit Kontakt zu Tuberkuloseerkrankten

    ( ) Ja ( ) Nein ( ) Unklar

8. **Wurde bei Ihnen eine der folgenden Erkrankungen festgestellt? (Bitte zutreffendes ankreuzen.)**

    ☐ HIV-Infektion/AIDS

    ☐ Diabetes mellitus (Zuckerkrankheit)

    ☐ Hepatitis A

    ☐ Hepatitis B

    ☐ Hepatitis C

    ☐ Nierenerkrankung

    ☐ Krebs

    ☐ Silikose

9. **Liegt bei Ihnen eine der folgenden Suchterkrankungen vor? (Bitte zutreffendes ankreuzen.)**

    ☐ Alkoholabhängigkeit

    ☐ Heroinabhängigkeit

    ☐ Tabakrauchen

    ☐ „Crack“ rauchen

    ☐ Methadon Substitution

10. **Haben Sie jemals intravenös Drogen konsumiert?**

    ( ) Ja ( ) Nein

11. Nehmen Sie regelmäßig Medikamente ein?

( ) Ja ( ) Nein,

wenn ja, welche? ___________________________

12. Leiden Sie aktuell unter folgenden Symptomen? (Bitte zutreffendes ankreuzen.)

□ Husten > 3 Wochen

□ Fieber

□ Nachtschweiß

□ Ungewollter Gewichtsverlust in den letzten 3 Monaten

□ Auswurf beim Husten

MULTIRESISTENTE ERREGER

13. Haben Sie in den letzten 4 Wochen Antibiotika eingenommen?

□ Nein

□ Ja, und zwar folgende Substanz(en): _____________________________

Im Zeitraum von _______ bis _________

14. Hatten Sie in den letzten 4 Wochen Haut- oder Weichgewebeinfektionen?

□ Nein

□ Ja

Vielen herzlichen Dank für die Teilnahme an unserer Studie!
Sollten Sie weitere Rückfragen haben, stehen wir Ihnen gerne zur Verfügung. Die Kontaktinformationen finden Sie auf dem Aufklärungsbogen, von dem Sie eine Kopie von uns erhalten haben.
