# Supplementary material for: Prevalence of latent tuberculosis in homeless persons: A single-centre cross-sectional study, Germany
Source: PLoS One. 2019 Mar 26;14(3):e0214556. doi: 10.1371/journal.pone.0214556 (PMC6435138; doi:10.1371/journal.pone.0214556)
Supplement: S2 File — (PDF) [file pone.0214556.s002.pdf]

Personal data

To be kept seperately

Study: Tuberculosis in homeless

Prevalence of latent infections with *Mycobacterium tuberculosis complex* in the homeless population of Münster

Study number: MTBW ______

1. **Date:**
2. **Person**
    a. Last name, first name: ____
    b. Date of birth: ___________________
    c. Contact details in case of positive testing:

        Phone: ____

        Whereabouts: _____________________________________

Case Report Form

Study: Tuberculosis in homeless

Prevalence of latent infections with *Mycobacterium tuberculosis complex* in the homeless population of Münster

Study number: MTBW ______

CRITERIA FOR INCLUSION

1. Declaration of consent
2. Minimum age: 18 years
3. Maintained legal capacity of contracting and capable of understanding
4. Subject speaks one of the languages, in which documents are available
5. Utilisation of assistance for homeless

CRITERIA FOR EXCLUSION

1. Are you pregnant? (As far as known.)

☐ Yes

☐ No

DEMOGRAPHICAL DATA

2. Date:

3. Person

a. Year of birth: ____________________
b. Place of birth: _______________________
c. Nationality: _______________________
d. Predominant place of residence in the past 5 years (country): ___________________
e. Have you ever been in jail?

☐ No

☐ Yes, country: __________________

f. Length of stay in the „Haus der Wohnungslosenhilfe“ up to now (days): _______

g. Sex

( ) m ( ) f ( ) other

h. Height (cm): _______________

i. Bodyweight (kg): ___________________

j. Have you been vaccinated against BCG?

( ) Yes ( ) No ( ) Unknown

4. Education/Job

a. How many years did you attend school?

______ years

b. Which occupation do you follow at the moment?

- ☐ _______________
- ☐ I am currently unemployed

c. Which professions have you pursued before?

____

____

5. How many months of your life (overall) have you been without a permanent residence?

- ☐ <6 months
- ☐ 6-24 months
- ☐ >24 months

6. State of insurance

- ☐ Statutory health insurance in Germany (*GKV*)
- ☐ Private health insurance in Germany
- ☐ Expenses covered by the *Sozialamt*
- ☐ Insured in the EU with claims in Germany
- ☐ Insured abroad (non EU) with claims in Germany
- ☐ No health insurance

TUBERCULOSIS

7. **Tuberculosis in the past**
   a. Have you been diagnosed with tuberculosis before?

      ( ) Yes ( ) No ( ) Unknown

   b. Have you ever been trated for tuberculosis?

      ( ) Yes ( ) No ( ) Unknown

   c. Have you ever had contact with persons suffering from tuberculosis ?

      ( ) Yes ( ) No ( ) Unknown

8. **Have you been diagnosed with one of the following diseases? (Please mark where applicable.)**

   □ Infection with HIV/AIDS

   □ Diabetes mellitus

   □ Hepatitis A

   □ Hepatitis B

   □ Hepatitis C

   □ Nephropathy (kidney disease)

   □ Cancer

   □ Silicosis

9. **Do you have an addictive disorder? (Please mark where applicable.)**

   □ Alcohol addiction

   □ Heroin addiction

   □ Smoking tobacco

   □ Smoking „crack“

10. **Have you ever consumed drugs intravenously?**

    ( ) Yes ( ) No

11. **Do you regularly take medicine?**

( ) Yes ( ) No ,

If yes, what kind? __________________________

b. Do you currently receive a substitution therapy due to an addictive disorder?

( ) Yes ( ) No ,

If yes, what kind? __________________________

12. Do you currently show the following symptoms? (Please mark where applicable.)

- ☐ Cough > 3 weeks
- ☐ Fever
- ☐ Night sweat
- ☐ Unintentional loss of weight within the last 3 months
- ☐ Cough with expectoration

MULTIRESISTANT PATHOGENS

13. Have you taken antibiotics within the last 4 weeks?

- ☐ No
- ☐ Yes, the following substance(s): ____________________________

In the period from _______ to _________

14. Have you had infections oft he skin or of soft tissue within the last 4 weeks?

- ☐ No
- ☐ Yes

Thank you very much for taking part in our study!
If you have any further questions, we are gladly availble. You can find our contact details on the consent form of which you received a copy.
