# Supplementary material for: Prevalence of latent tuberculosis in homeless persons: A single-centre cross-sectional study, Germany
Source: PLoS One. 2019 Mar 26;14(3):e0214556. doi: 10.1371/journal.pone.0214556 (PMC6435138; doi:10.1371/journal.pone.0214556)
Supplement: S1 Table — (DOCX) [file pone.0214556.s003.docx]

**S1 Table**: Characteristics of the study population and comparison of participants with and without a positive/borderline Interferon-γ release assay (IGRA)

|  |  | Total (n=142) | IGRA negative (n=119) | IGRA positive/borderline (n=23) | OR (95%CI) | p-value |
| --- | --- | --- | --- | --- | --- | --- |
| Demographic data | Mean age [years] (SD) | 42.4 (12) | 41.9 (12) | 45.0 (15) | NA | 0.4 |
|  | Sex [female] | 14 (10) | 12 (10.1) | 2 (9) | 0.9 (0.2–4.1) | 1 |
|  | Median BMI [kg/m^2^] (range) | 24.4 (17.2−40.1) | 23.9 (17.2−40.1) | 24.5 (17.9−37.2) | NA | 1 |
|  | Health insurance | 119 (84) | 103 (87) | 16 (70) | 2.8 (1−7.9) | 0.04 |
| Citizenship | Bulgarian | 9 (6) | 4 (3) | 5 (22) | Reference | - |
|  | German | 83 (58) | 75 (63) | 8 (35) | 0.1 (0.02−0.4) | 0.01 |
|  | Polish | 12 (8) | 10 (8) | 2 (9) | 0.2 (0.02−1.2) | 0.1 |
|  | Slovak | 9 (6) | 7 (6) | 2 (9) | 0.2 (0.03−1.8) | 0.2 |
|  | Other^a^ | 29 (20) | 23 (19) | 6 (26) | 0.2 (0.04−1.0) | 0.05 |
| Country of birth | Bulgaria | 9 (6) | 4 (3) | 5 (22) | Reference | - |
|  | Germany | 73 (51) | 68 (57) | 5 (22) | 0.06 (0.01−0.3) | <0.001 |
|  | Poland | 13 (9) | 11 (9) | 2 (9) | 0.2 (0.02−1.1) | 0.06 |
|  | Other^b^ | 47 (33) | 36 (30) | 11 (48) | 0.2 (0.1−1.1) | 0.06 |
| Country of main residence in the past 5 years | Germany | 114 (80) | 98 (82) | 16 (67) | 0.04 (0−0.4) | 0.005 |
|  | Bulgaria | 5 (4) | 1 (1) | 4 (17) | Reference | - |
|  | Other^c^ | 23 (16) | 20 (17) | 3 (13) | 0.04 (0−0.5) | 0.01 |
| Exposure to TB^d^ | Citizen of a low-incidence country | 98 (70) | 87 (74) | 11 (48) | 0.3 (0.1−0.8) | 0.01 |
|  | Born in a low-incidence country | 90 (64) | 83 (70) | 7 (30) | 0.2 (0.1−0.5) | <0.001 |
|  | Main residence in a low-incidence country in the past 5 years | 127 (93) | 111 (96) | 16(76) | 0.1 (0.04−0.6) | 0.002 |
| Overall duration of homelessness during lifetime | 6−24 months | 30 (21) | 27 (23) | 3 (13) | Reference | - |
|  | <6 months | 46 (33) | 40 (34) | 6 (26) | 1.4 (0.3−5.9) | 0.7 |
|  | >24 months | 65 (46) | 51 (43) | 14 (61) | 2.5 (0.7−9.4) | 0.2 |
| Duration of current stay at shelter in Münster | Median duration [days] (range) | 28 (0−3650) | 28 (0−3650) | 26 (0−2190) | NA | 0.9 |
|  | Imprisonment in the past | 73 (52) | 62 (53) | 11 (48) | 1.2 (0.5−3) | 0.7 |
| Education/employment | Median years of school attendance (range) | 10 (0–20) | 10 (0−20) | 9 (0−12) | NA | 0.006 |
|  | Completed apprenticeship | 64 (53) | 56 (55) | 8 (44) | 1.5 (0.6−4.2) | 0.4 |
|  | Currently unemployed | 92 (71) | 76 (70) | 16 (76) | 0.7 (0.3−2.2) | 0.6 |
|  | Former and current employment in medical sector | 14 (12) | 13 (12) | 1 (5) | 2.7 (0.3−21.5) | 0.7 |
|  | Former and current employment as craftsman | 68 (54) | 55 (52) | 13 (65) | 0.6 (0.2−1.6) | 0.3 |
|  | Former and current employment in gastronomy | 28 (22) | 23 (22) | 5 (25) | 0.8 (0.3−2.5) | 0.8 |
|  | Former and current employment in other sectors | 77 (59) | 66 (61) | 11 (52) | 1.4 (0.6−3.6) | 0.5 |
| Medical history | BCG vaccination | 23 (24) | 21 (25) | 2 (14) | 2 (0.4−9.8) | 0.5 |
|  | Known TB infection in the past | 5 (4) | 4 (3) | 1 (4) | 0.8 (0.1−7.2) | 1 |
|  | Contact with TB patients in the past | 16 (12) | 15 (14) | 1 (5) | 3.4 (0.4−27.4) | 0.3 |
|  | Any of the recorded chronic diseases (below) | 31 (22) | 26 (22) | 5 (22) | 1 (0.3−3) | 1 |
|  | Diabetes mellitus | 11 (8) | 10 (8) | 1 (4) | 2 (0.3−16.6) | 0.5 |
|  | Cancer | 4 (3) | 3 (3) | 1 (4) | 0.6 (0.1−5.7) | 0.5 |
|  | Hepatitis C | 11 (8) | 8 (7) | 3 (13) | 0.5 (0.1−2) | 0.4 |
|  | HIV | 0 (0) | 0 (0) | 0 (0) | NA | NA |
| Signs/symptoms of TB | Cough for more than 3 weeks | 38 (27) | 28 (24) | 10 (44) | 0.4 (0.2−1) | 0.05 |
|  | Fever | 12 (8) | 9 (8) | 3 (13) | 0.6 (0.1−2.2) | 0.4 |
|  | Night sweat | 17 (12) | 16 (13) | 1 (4) | 3.4 (0.4−27.1) | 0.3 |
|  | Weight loss in the past 3 months | 11 (8) | 10 (8) | 1 (4) | 2 (0.3−16.6) | 1 |
|  | Production of sputum when coughing | 42 (30) | 34 (29) | 8 (35) | 0.8 (0.3−1.9) | 0.6 |
| Drug addiction | Any of the recorded addictions (below) | 123 (87) | 101 (85) | 22 (96) | 0.3 (0.03−2) | 0.2 |
|  | Alcohol | 41 (29) | 37 (31) | 4 (17) | 2.1 (0.7−6.7) | 0.2 |
|  | Nicotine | 117 (82) | 96 (81) | 21 (91) | 0.4 (0.1−1.8) | 0.2 |
|  | Heroin | 19 (13) | 14 (12) | 5 (22) | 0.5 (0.2−1.5) | 0.2 |
|  | Cocaine (smoking) | 9 (6) | 8 (7) | 1 (4) | 1.6 (0.2−13.3) | 1 |
|  | i.v. drug abuse any time | 25 (18) | 21 (18) | 4 (17) | 1 (0.3−3.3) | 1.0 |
|  | Substitution treatment | 12 (8) | 8 (7) | 4 (17) | 0.3 (0.1−1.3) | 0.1 |
| Medication | Any regular medication | 54 (38) | 45 (38) | 9 (39) | 1 (0.4−2.4) | 0.9 |
|  | Psychotropic drugs | 26 (19) | 23 (20) | 3 (13) | 1.7 (0.5−6.1) | 0.6 |
|  | Antihypertensive Drugs | 10 (7) | 9 (8) | 1 (4) | 1.9 (0.2−15.5) | 1 |
|  | Medication for diabetes mellitus | 9 (7) | 8 (7) | 1 (4) | 1.6 (0.2−13.8) | 1 |
|  | Analgesic drugs | 8 (6) | 7 (6) | 1 (4) | 1.4 (0.2−12.1) | 1 |
|  | Gastric protection drugs | 8 (6) | 8 (7) | 0 (0) | NA | 0.4 |
|  | Other drugs | 8 (6) | 5 (4) | 3 (13) | 0.3 (0.1−1.4) | 0.1 |

NB: All values are n (% of group – Total/IGRA negative/IGRA positive) unless indicated otherwise. NA=not applicable

^a^ Other citizenships: Austrian (n=1), Congolese (n=1), Croatian (n=1) Ethiopian (n=1), Greek (n=1), Guinea-Conakrian (n=1), Hungarian (n=1), Iran (n=1), Italian (n=3), Kosovan (n=1), Latvian (n=1), Lithuanian (n=2), Nigerian (n=1), Portuguese (n=1), Romanian (n=3), Russian (n=1), Senegalese (n=1), Serbian (n=1), Somalian (n=1), Syrian (n=2), Turkish (n=3)

^b^ Other countries of birth: Azerbaidschan (n=1), Congo (n=1), Croatia (n=1), Ethiopia (n=1), Eritrea (n=1), Gambia (n=1), Greece (n=1), Guinea-Conakry (n=1), Hungary (n=1), India (n=1), Iran (n=1), Iraq (n=2), Israel (n=1), Italy (n=3), Kenia (n=1), Kosovo (n=1), Latvia (n=1), Lithuania (n=2), Netherlands (n=1), Portugal (n=1), Romania (n=3), Russia (n=1), Senegal (n=1), Serbia (n=1), Somalia (n=1), Syria (n=3), Turkey (n=4)

^c^ Other countries of main residence in the past five years: Croatia (n=1), Ethiopia (n=1), travelled in European Union (n=5), France (n=1), Greece (n=1), Hungary (n=1), Italy (n=2), Netherlands (n=1), Poland (n=1), Slovakia (n=3), Spain (n=1), Syria (n=2), USA (n=1), no data (n=1)

^d^ Low-incidence countries for TB as defined by the World Health Organization (<100 cases per 100000 population)
